# Country perspectives on improving technical assistance in the health sector

**DOI:** 10.12688/gatesopenres.13248.1

**Published:** 2021-08-24

**Authors:** Natasha Kanagat, Jeanne Chauffour, Jean-Fidèle Ilunga, Sylvain Yuma Ramazani, John J.P. Ovuoraye Ajiwohwodoma, Salma Ibrahim Anas-Kolo, Oleka Maryjane, Nkeiru Onuekwusi, Toto Ezombe, James Dominion, Joseph Sunday, Joseph Kasongo, Gavial Ngambwa, Christy Asala, Célestin Nsibu, Abimbola Williams, Melanie Wendland, Emilia Klimiuk, Anne LaFond, Nosa Orobaton, Dyness Kasungami

**Affiliations:** 1 JSI Research & Training Institute, Inc., Arlington, VA, 22202, USA; 2 Ministry of Health of the Democratic Republic of the Congo, Kinshasa, Democratic Republic of the Congo; 3 Federal Ministry of Health, Federal Republic of Nigeria, Abuja, Nigeria; 4 National Primary Health Care Development Agency, Abuja, Nigeria; 5 Independent Consultant, Abuja, Nigeria; 6 Kaduna State Ministry of Health, Kaduna, Nigeria; 7 White Ribbon Alliance, Abuja, Nigeria; 8 Sonder Collective, Helsinki, Finland; 9 Bill & Melinda Gates Foundation, Seattle, WA, 98109, USA

**Keywords:** TA, technical assistance, MNCH, maternal health, neonatal health, child health, health systems strengthening, HCD, re-design, reimagine, re-imagine, redesign, human-centered design, co-creation, DRC, Nigeria, Democratic Republic of the Congo, Democratic Republic of Congo, maternal and child health

## Abstract

**Background:** This paper presents learnings from the Re-Imagining Technical Assistance for Maternal, Neonatal, and Child Health and Health Systems Strengthening (RTA) project implemented in the Democratic Republic of the Congo and Nigeria from April 2018 to September 2020 by JSI Research & Training Institute, Inc. and Sonder Collective and managed by the Child Health Task Force. The first of RTA’s two phases involved multiple design research activities, such as human-centered design and co-creation, while the second phase focused on secondary analysis of interviews and reports from the design research. This paper explores the limitations of current technical assistance (TA) approaches and maps opportunities to improve how TA is planned and delivered in the health sector.

**Methods:** We analyzed project reports and 68 interviews with TA funders, providers, and consumers to explore in greater detail their perspectives on TA, its characteristics and drawbacks as well as opportunities for improvement. We used qualitative content analysis techniques for this study.

**Results:** The issues surrounding TA included the focus on donor-driven agendas over country priorities, poor accountability between and within TA actors, inadequate skill transfer from TA providers to government TA consumers, an emphasis on quick fixes and short-term thinking, and inadequate governance mechanisms to oversee and manage TA. Consequently, health systems do not achieve the highest levels of resilience and autonomy.

**Conclusions:** Participants in project workshops and interviews called for a transformation in TA centered on a redistribution of power enabling governments to establish their health agendas in keeping with the issues that are of greatest importance to them, followed by collaboration with donors to develop TA interventions. Recommended improvements to the TA landscape in this paper include nine critical shifts, four domains of change, and 20 new guiding principles.

## Introduction

Technical assistance (TA) in the health sector has been defined and interpreted in myriad ways ^1– 4^. TA is generally characterized as short- or long-term support provided by one institution to another on a specific topic or topics. The support can be provided via training, seminars, or the transfer of knowledge through collaboration between institutions. TA is typically financed and designed by funding organizations like bilateral or multilateral agencies or private philanthropies. This type of TA aims to improve the effectiveness and efficiency of health systems and organizations. TA processes typically involve multiple actors and operate within a political, legal, and financial context. In this paper, we focus on external TA as a mechanism for health-focused technical and foreign assistance. This type of TA which is a structural mechanism through which a large portion of foreign assistance is delivered, is one of the four functions of the global health architecture framework as described by the Bill & Melinda Gates Foundation (BMGF), the others being agenda setting, finance, data, and monitoring ^5^.

Estimates of TA spending in the health sector as a proportion of foreign assistance vary depending on how TA and foreign assistance are defined and packaged ^6^. Foreign assistance spending is often reported as a single figure not disaggregated by the type of aid provided. For example, the Organisation for Economic Co-operation and Development (OECD) recorded US $19.2 billion in 2018 spending by OECD countries on technical cooperation, a term which includes grants and loans to recipient country nationals as well as funding for consultants who are working in recipient countries ^7^, while a Congressional Research Service report indicated that the U.S. alone spent $46.89 billion in foreign assistance programs in 2018, including military and security assistance ^8^. A 2011 Action Aid reported that about 33 percent of 2010 foreign assistance was spent on TA ^9^. Reports on general development assistance for health (DAH) ^1^ from the Institute for Health Metrics and Evaluation (IHME) reveal significant investment in the health sector since 1990, but the sources and mechanisms of funding and health priority areas have shifted over time. IHME estimates that in 2000 the U.S. total DAH was $12.4 billion; in 2015 it was around $37.9 billion; and in 2019, it was an estimated $40.6 billion ^10^. During this same period, government health spending and out-of-pocket health expenditures have increased; they are expected to continue on that trajectory.

Despite significant investments in DAH, by the end of the Millennium Development Goals period in 2015, most maternal, neonatal, and child health (MNCH) targets had not been achieved ^11^. Today there is concern that the world will not meet the Sustainable Development Goals (SDGs) for 2030 ^12^. As the global health community seeks to understand why these global targets remain elusive, TA approaches have come under increased scrutiny. Critics note that TA is often not aligned with country priorities or coordinated effectively with governments and other stakeholders. It is focused on short-term wins and lacks systematic approaches to solving public health challenges ^4^. Initiatives like the Paris Declaration on Aid Effectiveness (2005), the Accra Agenda for Action (2008) ^13^, and the Fourth High Level Forum on Aid Effectiveness in Busan (2011) ^14^ were established to improve the efficacy of TA. All these efforts called for better coordination between donors and countries, closer alignment of TA to country priorities, country ownership of TA, and the implementation of measurement and evaluation systems to ensure accountability among all parties. Donors were also urged to share information with each other and with governments to avoid duplication of programs and systems.

Both the Paris Declaration and the Accra Agenda included recommendations that TA providers make capacity building a fundamental feature of TA to enable countries to operate their health systems effectively and autonomously in the future. However, a 2011 evaluation of the Paris Declaration indicated that while the Principles provided a useful benchmark to guide foreign assistance, there continued to be poor alignment between foreign assistance and country priorities as outlined in national strategic development plans, donor performance was still not rigorously evaluated (thus hindering mutual accountability between donors and countries), and capacity development remained uncoordinated and its effectiveness unexamined, resulting in limited understanding of its efficacy ^15^.

In general, there is scant evidence on the effectiveness of TA and how it may contribute to improve health outcomes ^6, 16, 17^. In the absence of sound data on the effectiveness of TA approaches, the slow rate of reduction in maternal and child mortality maternal and child mortality are treated as proxies for the failures of TA. It is critical to increase efforts to assess the effectiveness of TA mechanisms to understand what does and does not work and use this knowledge to guide TA investments and approaches. The urgency of strengthening TA is heightened when infectious disease outbreaks like Ebola and coronavirus disease 2019 (COVID-19) strain domestic, regional, and global economies and public health systems, forcing countries to make difficult decisions about resource allocation and the use of TA resources.

### Purpose of this paper

The purpose of this paper is to present learnings from the Re-Imagining Technical Assistance for Maternal, Neonatal, and Child Health and Health Systems Strengthening (RTA) project implemented in the Democratic Republic of the Congo (DRC) and Nigeria from April 2018 to September 2020 by JSI Research & Training Institute, Inc. (JSI) and Sonder Collective (Sonder) and managed by the Child Health Task Force. The Child Health Task Force, created in 2017 and funded by the BMGF, is a network of global and country-based organizations and individuals working to design and implement equitable, comprehensive, and integrated child health programs using a life-course approach to achieve better outcomes for children ^18^.

RTA’s purpose was to identify the shortcomings of existing TA approaches and clarify opportunities for planning and delivering TA more effectively. Project strategy included co-creating a shared vision for improving TA and developing a model/roadmap using a human-centered design (HCD) and systems-design approach. Complementary publications from RTA further outline the project methods ^19– 22^. The project included two phases, the first of which featured several design research activities, including HCD and co-creation processes, while the latter focused on secondary analysis of interviews and reports from the design research.

This paper seeks to answer the following questions:

- What are the defining characteristics of TA?
- What are the issues associated with how TA is currently designed and implemented?
- What opportunity areas for change did project participants identify?

### The RTA project

The RTA project engaged with participants representing organizations that fund TA in the DRC and Nigeria health sectors (TA donors and funders), organizations that are funded by donors to implement TA in the health sector (TA providers), and government and health sector representatives who engage with donors and providers and use or benefit from the financial and non-financial resources associated with TA (TA consumers). Some organizations play dual roles; for example, multilateral organizations like the World Health Organization (WHO) and UNICEF can be TA providers and also fund other organizations that provide TA. In the DRC, TA donors such as the World Bank, the European Union, and the Belgian Cooperation Agency (CTB) have also served as TA providers. In some cases, the national government provides TA to organizations at the subnational level. RTA focused on TA providers funded by external donors. (When TA funder, donors, providers, and consumers are mentioned in the rest of this paper we use the definitions above. TA actors is a broader term encompassing funders, donors, providers, and consumers.)

The DRC and Nigeria were purposefully selected as implementation partners because their national MNCH programs are long-standing consumers of TA on MNCH issues. UNICEF considers the DRC a “high maternal mortality country” and Nigeria a “very high maternal mortality country.” ^23^ Both countries also receive considerable DAH from bilateral and multilateral donors ^24^. In addition, JSI had a history of partnering with multiple organizations in both countries including their ministries of health (MOH) on their national MNCH programs. Finally, JSI staff were available in the DRC and Nigeria to support RTA.

## Methods

### Data collection

***Data sources.*** This manuscript draws on four project reports and 68 interviews conducted as part of RTA’s HCD approach, described below. We expand on the analysis approach after providing background on the HCD and co-creation processes that constituted the data collection for this project.

***Human-centered design and co-creation processes.*** Sonder and JSI facilitated a series of HCD and co-creation processes with individuals in Nigeria and the DRC who have participated in or received TA ^19– 20^. This included interviews and workshops to gain a deep understanding of participants’ experiences with TA, reflect on opportunity areas for addressing the issues associated with existing TA practices, and ideate on potential ways to re-imagine TA.

***Workshops and participant selection.*** To ensure that the project was firmly rooted in TA experiences in both countries, individuals representing institutions that funded, provided, or sought TA in each country were invited to form a co-creation team to center their TA experiences and ensure ownership of the ideas that would be generated ^19^. RTA held three workshops in Nigeria and four in the DRC ^19^. Workshops provided a space for participants to share and reflect on their experiences with TA and ways in which it which could be strengthened. Each co-creation team comprised a dozen people who were purposefully selected to represent the federal, state/provincial, or subnational governments, civil society, non-governmental organization (NGOs), and universities. Co-creation team members received reimbursement for their transportation and all workshops included a complementary lunch, morning refreshments, and afternoon coffee. Team members were not paid for accommodation or meals. Detailed field notes were taken both during and after workshops.

***Interviews and participant selection.*** As part of the HCD process, the team also conducted 68 one-on-one interviews with individuals representing TA donors and funders, consumers, and providers. The objective of these interviews was to understand the current challenges and future solutions for TA by different TA actors based on the roles they played in the TA landscape. Interview participants were selected using a combination of purposive, convenience, sampling. We first created a list of potential participants organized by TA actor category and involvement in the DRC and Nigeria health sectors. Participants were then approached face-to-face, by telephone, email, and LinkedIn. Interviews were conducted in participants’ workplaces or conference centers where health-sector meetings were being held in Kinshasa and Abuja. Interviews were conducted using prompts and lasted between 30 and 120 minutes. Interviewers and participants were the only people present during interviews. Detailed notes were taken both during and after interviews. All study materials can be found as extended data ^25^.

***Data processing.*** After each workshop and interview, project staff transcribed verbatim what participants had said and identified topics to use as prompts and guide discussion during subsequent workshops ^19^. These findings were frequently verified with participants to obtain feedback before the next workshop. Data collection ceased once data saturation was achieved. DRC and Nigeria data were analyzed separately, then themes common to both countries were identified.

All interviews in the DRC were conducted in French by fluent French speakers. To ensure that the integrity of the conversations and the context were maintained, the interviews were then transcribed and translated into English by a French-speaking researcher who was present during the interview.

By the end of the final workshop in each country, participants had prioritized the design principles and concepts that would inform their roadmaps for change. The roadmaps consisted of critical shifts needed to transform the current state of TA into an improved future state. The critical shifts were summarized into four broad domains of change (i.e., strengthen the system as a whole, foster strong governance, build on the existing system, and cultivate trust). Each domain of change was then associated with five design principles that captured the underlying issues identified in the workshops and interviews as well as related recommendations for actions to improve TA.

RTA’s findings and the roadmap of critical shifts, domains of change, and design principles were presented during a one-day integration meeting that brought together a wider audience, including TA providers, donors, and national and state/provincial representatives who had not participated in the previous workshops. Sonder and JSI produced individual country case studies, an anthropological report on the DRC, and a summary report on both countries ^19– 22^.

***Consent procedures.*** Verbal consent was sought from all participants during interviews and workshops. They were assured of confidentiality and that all findings would be anonymized. The RTA project received approval from the MOH in both countries to conduct the workshops and interviews. Ethical approval from an institutional review board was not sought since the study was deemed to carry minimal risk for participants given the topic.

### Analysis

***Secondary analysis of interviews and project reports.*** For this manuscript, two researchers re-analyzed 68 interviews (29 in the DRC and 39 in Nigeria), two country case studies ^19– 20^, an anthropological report on the DRC ^21^, and the summary report on both countries ^22^ to more deeply explore participants’ perspectives on TA, its characteristics and limitations, and roadmaps for change. We applied qualitative content analysis techniques to examine the interview transcripts and project reports.

***Interviews.*** To standardize analysis and synthesis of the interviews, we created a codebook with a list of codes and code definitions to be applied to all data. The codes, developed based on the three key questions listed earlier, were created prior to the analyses of interviews for this paper. The codebook was transferred to a Google Sheet to enable access from various locations since the authors of this manuscript were working remotely. We did not use any software for the analysis.

Interviews from each country were analyzed and coded separately in the Google Sheet to ensure that country-specific contextual findings were not overlooked. We copied relevant text from the transcripts to the Google Sheet under the relevant codes. We also drew on quotes from the transcripts to illustrate the major themes and reviewed products and reports to verify our findings. Staff analyzing the data met frequently to discuss the codes, confirm their consistent application, and identify themes specific to each country and common across Nigeria and the DRC. In the event that there was inconsistent application of codes, both researchers met to discuss the text under consideration as well as the code definitions and ensured a standard application of codes. The themes identified in this manuscript were derived from the data. Since participants had reviewed and provided feedback on the project reports and validated findings during workshops, this manuscript was not shared back with them for another round of review.

***Project reports.*** All project reports were re-read to extract data based on the codes in the Google Sheet used for the interview data analysis. The intent behind analyzing reports was to complement the findings from the interview analyses by identifying themes that were common to those identified in the interviews as well as themes that offered an alternative explanation.

## Results

Below we present participant perceptions of TA in the DRC and Nigeria health sectors, beginning with how they defined and characterized TA. Then we report on the shortcomings of TA that were identified through the thematic analysis, and finally, we describe critical shifts needed in TA approaches and strategies to address the shortcomings.

### TA definitions and characteristics

Participants acknowledged that there is no commonly accepted definition of TA in the DRC or Nigeria. They broadly defined TA as the engagement of domestic or international expertise to address a gap in one or more functions of the health sector. Participants mentioned that TA can draw on a wide range of subject-area expertise, such as logistics and supply chain processes, technical program design, operations management, and administrative systems. TA was described as short- or long-term, and TA actors comprised funders, donors, providers, and consumers (see earlier definitions of these terms).

**Figure 1.**
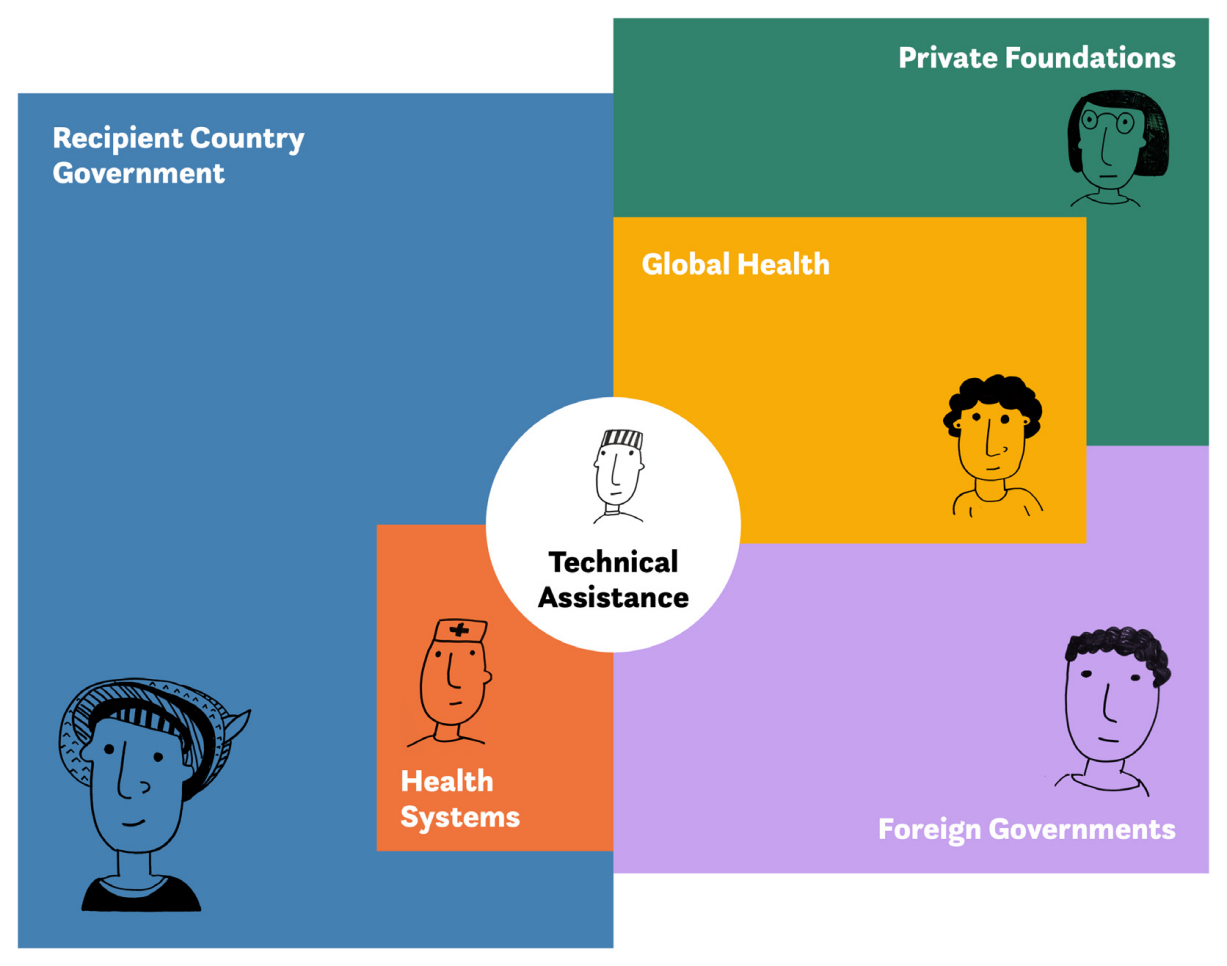
Technical assistance actors, services, and interactions. Note: Has been reproduced with permission from Sonder Collective, 2020 ^22^.

Workshop and interview participants noted that TA can be executed in parallel to a national health system or be integrated into the health system. Participants said that TA is a development mechanism that operates in a complex, dynamic system of actors, services, and interactions. They also pointed out that TA is carried out in, and interacts with, multifaceted political, financial, academic, and scientific systems, including country governments and health systems, private foundations, development agencies affiliated with country governments, and the broad global health network of academic institutions and NGOs. A change in one of the systems or organizations affects how TA is designed and consumed. (see Figure 1 for an illustration of the many interactions that occur through TA).

### Limitations and implications of existing TA practices

***Donor-driven TA agendas and country needs and priorities.*** In both countries, participants stated that TA is usually initiated by donors. In many cases, donor agendas do not seem to align with the country’s priorities as described in its national health development or strategic plans. Because TA often does not address country priorities, participants reported that TA consumers often feel that their voices are not heard and their needs are not met. Participants noted that TA providers do not seem to have a genuine interest in partnering with them to address country-defined issues in ways that emphasize the long-term improvement of population health (e.g., building local knowledge and expertise, defining impact measures).

***“A partner comes in, does his project, identifies the needs, hires the technical assistant in an attempt to work more closely with the government, and places this technical assistant within the MOH. That is the first problem: in the identification of needs, TA is externalized, it is not internalized.”***

***- TA Donor/Provider, DRC***

Government participants shared that they often feel compelled to accept foreign aid, even if the proposed TA does not align with the country’s strategic objectives. They feel that their country is not in a position to refuse assistance that could improve any aspect of the health sector, even if the intervention itself is not deemed essential. To ensure that TA is aligned with country priorities, participants encouraged TA providers to collaborate with other TA actors, be flexible in allocating funds, and convey respect for the government’s authority. Government participants also acknowledged that, to build TA providers’ trust in the government’s ability to manage TA donors and providers, government actors should demonstrate leadership and direct, coordinate, and manage the TA delivered to their programs and agencies.

TA providers and consumers stated that misalignment between country needs and TA begins at the proposal stage when donors issue requests for proposals (RFP) that reflect partially designed projects. TA providers stated that because RFPs contain incomplete project designs and require a rapid submission of proposals, providers sometimes fail to fully research the country priorities or contexts, reverting to interventions, methods, and tools that have been effective in other settings. When TA providers conduct in-country research after their proposal is approved, they often believe they cannot refine the focus of the project at that point. Thus, direct TA support is often confined to the objectives in the TA provider’s terms of reference from the funder, with providers reporting being unable to pivot to respond to evolving situations and emerging needs. TA funders, providers, and consumers alike acknowledged that not permitting TA providers to modify their approach as needed is a critical flaw, and that steps are needed to improve how and when TA is tailored to a country’s context.

***“As an implementing partner, we discovered we have designed programs that do not respond to needs and because the donor does not have flexibility, we are forced to implement the activity without change.”***

***- TA Provider, Nigeria***

Participants also cited vertical programs like HIV/AIDS and immunization as examples of funder—rather than country—priorities dictating which health areas receive TA. They also noted that vertical programs result in the creation of duplicate data collection systems that do not strengthen primary health care and hamper program sustainability.

***Short-term TA and static, one-size-fits-all solutions.*** Participants expressed the belief that TA projects are generally not designed to bring about meaningful, lasting change, given that donor funding cycles necessitate rushed TA implementation. Participants agreed that while emergency and humanitarian crises require quick-moving, nimble TA, non-emergency TA designed to strengthen systems requires a long-term vision. Nevertheless, most TA initiatives are three-to-five years long, which participants stated is too brief to effect sustained change.

Participants commented that the prevailing approach to TA emphasizes short-term measures of progress rather than systems building. They cited the emphasis on output indicators (e.g., the number of people trained to deliver a given service) as opposed to measures that indicate changes in a system’s capacity to provide services over time. TA consumers and providers alike expressed concern that individuals and organizations that fund or provide TA benefit more from short-term projects than TA consumers do, which further undermines country priorities.

When a TA provider meets short-term, donor-defined outputs, they may increase the probability of securing additional work from that funder. Pressure to implement TA rapidly encourages providers to implement one-size-fits-all solutions that are not responsive to the context. TA providers stated that they feel constrained to produce reports and demonstrate evidence of impact quickly, necessitating parallel data collection systems to substitute for slow-moving national information and reporting systems. This approach does not contribute to systems strengthening or enhance the capacity of country-led health sectors.

Participants also recommended that governments counter the short-term nature of TA by becoming directly involved in TA program design and implementation, thereby enhancing local institutional memory and increasing the likelihood of sustained impact, scale-up, or adoption of the initiative by the government. As one participant stated, governments should be engaged in and familiar with health programs, and thoroughly document needs assessments and rollout processes. This would result in more sustainable TA activities rather than being aligned with one individual in the health system and that are unaffected by changes in government leadership.

***“[The time between] the formulation of the projects and their implementation can sometimes last years. At the central level, there can be some important changes. . . changes in ministers, etc. that mean that if we [TA donor/provider] do not have representation of TA at the central level, there is a loss of visibility. And the people who have participated at the central [government] level in the identification of needs are no longer there, and what is done locally is completely ignored by the incoming parties.”***

***- TA Donor/Provider, DRC***

***Insufficient skill transfer and dependencies.*** The practice of TA-supported interventions was described by participants as being “capacity filling” instead of “capacity building.” For example, when TA providers embed their staff in a MOH, they often neglect to allocate sufficient resources to strengthen the capabilities of MOH staff, which could enhance long-term sustainability. Many participants also criticized the attitude of certain TA providers who come to “do” the work themselves rather than assist, support, collaborate with, or learn from local partners. Participants concluded that this practice creates an over-reliance on donors and TA providers to conduct core government business and fails to leverage opportunities for local capacity development.

Participants explained that ideally, capacity building should be part of all TA contracts so that skills transfer is integrated into projects. They noted that an embedded approach, with TA providers working side-by-side and mentoring government staff, would ensure greater ownership by governments. Government staff would not feel excluded from learning the information and skills being used by TA providers and, consequently, would be better able to sustain the system after the TA ended. Participants also emphasized that capacity building is not a unidirectional effort and that TA providers would benefit from listening to the staff they train since staff often have a deep understanding of the nuances of the health system. Understanding these nuances would strengthen TA providers’ approach to capacity building, making their efforts more contextually relevant. Good TA through intentional capacity building signals an intent to invest in the system for long-term sustainability.

***“[Good TA should be a] passing [on] or transfer of skills and knowledge to those who don’t have it in a sustainable manner. When you are done, the people you have worked with will be able to carry on without you. They will be able to plan and make sure they meet their objectives.”***

***- TA Provider, Nigeria***

Government representatives reported that they have lost talented civil servants who left to work for development agencies to benefit from opportunities for professional growth and higher salaries. This loss of talent diminishes governments’ ability to enhance and sustain leadership capacity and project management skills due to brain drain and overreliance on external TA. Furthermore, participants said that the motivation and morale of government staff often suffer when local capacity is compared to, pitted against, or replaced by private-sector TA providers. Respondents shared that some civil servants grow reluctant to engage fully in the work they share with TA providers for fear of relinquishing their responsibilities and assignments. This hesitancy to collaborate with TA providers can contribute to growing gaps in institutional capacity and perpetuate reliance on external support.

Short-term TA investments routinely divert attention from issues of government ownership, institutional capacity building, and systems sustainability. When capacity building is not emphasized and sustained, as new projects are funded, the government continues to rely on external expertise rather than leading from within.

***Limited mutual accountability and uncoordinated TA.*** Government participants stated that there is limited or no obligation for TA funders or providers to interact and consult with governments, update them on progress, or share reports and evidence of their work. Government participants are often not informed about donors’ decisions related to selecting, funding, setting priorities for, and choosing a physical location for TA providers. In some cases, TA providers bypass national governments and contract directly with state or provincial governments, demonstrating their lack of accountability to national governments and further exacerbating government efforts to coordinate and manage TA.

Government participants acknowledged their critical role in coordinating TA, communicating their vision and expectations to TA providers and donors, and also highlighted the need for communication mechanisms through which they can receive updates on TA progress. They stated, however, that they are often unable to play a TA coordinating role because of competing priorities and their busy schedules. Government participants also felt that TA donors’ and providers’ rapid requests for input—when they do occur—are often not easy for them to meet as a result of their many job responsibilities. Participants representing governments also described the disconnect that occurs when donors and TA providers bring to bear resources that shift the balance of power and result in government staff feeling ineffectual and peripheral to the programs implemented in their country.

***“[We] can’t hold them [TA providers] responsible because we are not the ones funding them. [The] accountability is between them and their donors, because they had MOUs [memoranda of understanding] that did not include the country.”***

***– Government representative, Nigeria***

Government participants from the DRC and Nigeria described the challenges of coordinating different donor agencies, each with its own norms, financing processes, timelines, and expectations—especially given that many donors compete with one another and are reluctant to compromise or collaborate. With limited coordination and accountability among donors, at times, different TA providers implement projects with a similar purpose in the same location, using parallel data collection and reporting systems. In this situation, with multiple donors and TA providers intensely focused on a given health issue or geographic location, other health issues and locations are typically deprived of resources and expertise. TA providers emphasized the importance of governments closely reviewing TA providers’ terms of reference to coordinate TA and hold both funders and TA providers accountable.

Government participants also identified coordination mechanisms that frequently do not function as planned. For example, project approvals are often not communicated across government departments and at different levels of the government. Participants from the sub-national level shared that it is often unclear how funding decisions are made, with concerns that sub-national-level organizations receive a very small portion of TA funds for their programs. This causes mistrust about how resource-allocation decisions are made at the national level. In addition, according to sub-national level staff, the funding they received was insufficient to implement their programs. Participants noted the importance of government departments improving communication about TA funding streams and dollars allocated, along with the rationale for funding decisions.

In Nigeria, participants highlighted problems with coordination between state and federal governments that result from a decentralized health system and the growing autonomy of state governments. They did share the following best practice, which they hoped to scale to all partners: a memorandum of understanding that was signed between a funder, a TA provider, and the government to ensure mutual accountability and a more coordinated flow of information. Participants also mentioned the benefits of having technical working groups at the national level that coordinate strategies and activities for health programs.

Participants in the DRC highlighted several systemic accountability challenges, the first of which is the difficulty of tracking donor funds allocated and spent in the health sector. This lack of transparency contributes to TA providers’ distrust of the government. They also described a lack of clear communication and consensus on the TA to be done, the results and targets to be reached, and the benchmarks and deliverables to be met, suggesting the need for additional oversight and monitoring by the government and donors.

***Budget management in the health sector.*** In the DRC, participants had differing perspectives on the management of and channels for donor health sector funding. Donors and TA providers expressed reluctance to allow funds to be channeled through the health department’s budget, fearing that funds would be disbursed at the central level without reaching provinces, zones, and communities. TA providers also felt that providing direct budgetary support requires them to relinquish some decision-making power regarding how, where, when, and for what purpose funds are allocated and distributed. TA providers said that they prefer to manage and disburse funds for salaries and activities or send funds directly to on-the-ground decentralized intermediaries, such as local NGOs. Participants suggested that if perceptions of the government’s reliability in managing health-sector spending improved, TA providers would be more inclined to equip, support, and trust a central-level mechanism for disbursing funds, which would help to reduce the fragmentation that comes with programs being implemented simultaneously by multiple TA providers.

***Imbalances of power between government actors, TA providers, and funders.*** The findings above focus primarily on the mechanics of TA. Underlying these operational challenges are the cultural aspects of TA such as power imbalances—a fundamental driver of TA. The power dynamics between TA actors influence the way TA is managed, funded, designed, and implemented. This section draws on interviews and the Sonder DRC anthropological report ^21^, which closely considered the power relationships between TA actors, among other themes. While power dynamics were not examined as exhaustively in Nigeria, the overarching theme of power appeared in multiple ways, including the influence of power on interactions, priority setting, relationships, and decision making within and among groups of TA actors.

Respondents commented that donors have power because they provide the funding for activities and determine who receives funding and who does not. In countries with limited resources, if a donor provides funding for a health area that does not align with country priorities, those funds will not be declined given the urgent needs. With the DRC’s heavy reliance on donor funding, government participants said they find it very difficult to express a difference of opinion about donor and provider priorities because of the acute need for financial and technical support.

***“This situation puts the MOH in a situation of fragility because, since the technical assistance provider is the one bringing in the money, they end up being the one calling the shots. If the technical assistance provider says they do not agree, everyone changes their ways; if they say they agree, everyone else agrees too.”***

***- Government representative DRC***

TA providers are considered powerful, either because funding is channeled through them or the acquisition of funding is contingent on drawing on providers’ expertise. In theory, governments should be able to approve or end TA activities. Respondents also noted that government actors’ sense of powerlessness is further exacerbated when decision making and money are inextricably linked—for example, when TA providers demonstrate their allegiance and accountability to funders, reporting to funders before communicating with the government.

Participants also commented that power dynamics manifest in the flow of information between TA actors. Provincial and state government staff felt they are typically not invited by the MOH and other national agencies to participate in critical discussions, which results in their being left out of decisions. This gatekeeping of information erodes trust among TA actors and creates inefficiencies in health systems operations.

Another result of this power imbalance driven by a lack of transparency in financial resource flows, information sharing, and work assignments was feelings of frustration and demotivation among TA consumers, particularly government employees who felt their efforts were futile or unappreciated.

Critically, navigating these power dynamics distracted TA actors from fully turning their attention to the communities that were intended to be the focus of health care services.

***Summary of limitations of existing TA approaches.*** The leading TA issues that surfaced during interviews in the DRC and Nigeria included the focus on donor-driven agendas over country priorities, poor accountability within and among TA actors, inadequate transfer of skills from TA providers to governments, an emphasis on quick fixes and short-term thinking, and inadequate government mechanisms to oversee and manage TA—all of which combine to contribute to health systems that lack the authority, resources, and capacities to be resilient and function autonomously. Additionally, participants stated that it was unclear who benefits from TA and what the incentives are for TA actors to implement projects. The questioning of TA actors’ motivations, skewed power dynamics, and lack of accountability for TA result in a deficit of trust between TA actors and serve to intensify the challenges of working together in partnership.

Participants called for a shift in TA approaches and a redistribution of power so that governments can set their own health agendas, identify the issues that are of greatest importance to them, work with donors to design interventions, request TA support, and participate in the selection of TA providers. Additionally, participants also stressed the importance of establishing systems that allow governments to hold TA providers and donors accountable and government systems that promote transparency, responsibility, and a shared commitment to improving the health of communities.

### Guiding principles and critical shifts to improve TA

Participants identified nine critical shifts needed to transform current TA approaches into more sustainable, country-driven processes. These shifts, presented in Table 1, create a bridge between the TA shortcomings identified and the improved TA model proposed by the DRC and Nigeria co-creation teams and respondents. The shifts also describe the institutional changes required of all actors across the TA landscape.

**Table 1.** Critical shifts to transform technical assistance.

**Existing TA is...**	**Good TA should be...**	**The critical shifts**
**Donor-driven**	**Country-driven and** ** owned**	Shift away from a system where priorities are imposed by donors on countries to one where governments practice strong governance by leading TA agenda setting and coordination.
**Creating dependencies**	**Cultivating of self-** **reliance and autonomy**	Shift away from a system that depends on continuous donor support for survival to one that prioritizes self-reliance and autonomy by building on and strengthening the existing system.
**Lacking trust in** ** institutions and ** **individuals' motivations**	**Trust building**	Shift away from a system that perpetuates mistrust of institutions and individuals’ motivations to a more transparent and accountable environment where actors trust each other's motivations and approaches.
**Unaccountable**	**Accountable**	Shift away from a system where power structures and roles are vague and actions are rarely tied to consequences to one that fosters transparency and where individual actors are held accountable for their actions.
**Fragmented**	**Holistic**	Shift away from siloed, uncoordinated projects to collaborative, comprehensive, holistic initiatives.
**Supply-driven**	**Problem-focused**	Shift away from a system that simply allocates the available resources to one that assesses needs, determines the resources needed to fill gaps and address issues, and works toward acquiring the resources.
**Short-term**	**Sustainable and** ** resilient**	Shift away from a system that invests in quick fixes to one that prioritizes larger long-term gains in health sector outcomes and health system resiliency.
**Static**	**Adaptable**	Shift away from a static system to a flexible one that monitors, evaluates, quickly responds to data, and iterates as needed.
**Uprooted**	**Contextualized**	Shift away from a one-size-fits-all approach to problem solving to one that considers the local context and has the flexibility to adjust.

Note: Has been reproduced with permission from Sonder Collective, 2020 ^22^

**Figure 2.**
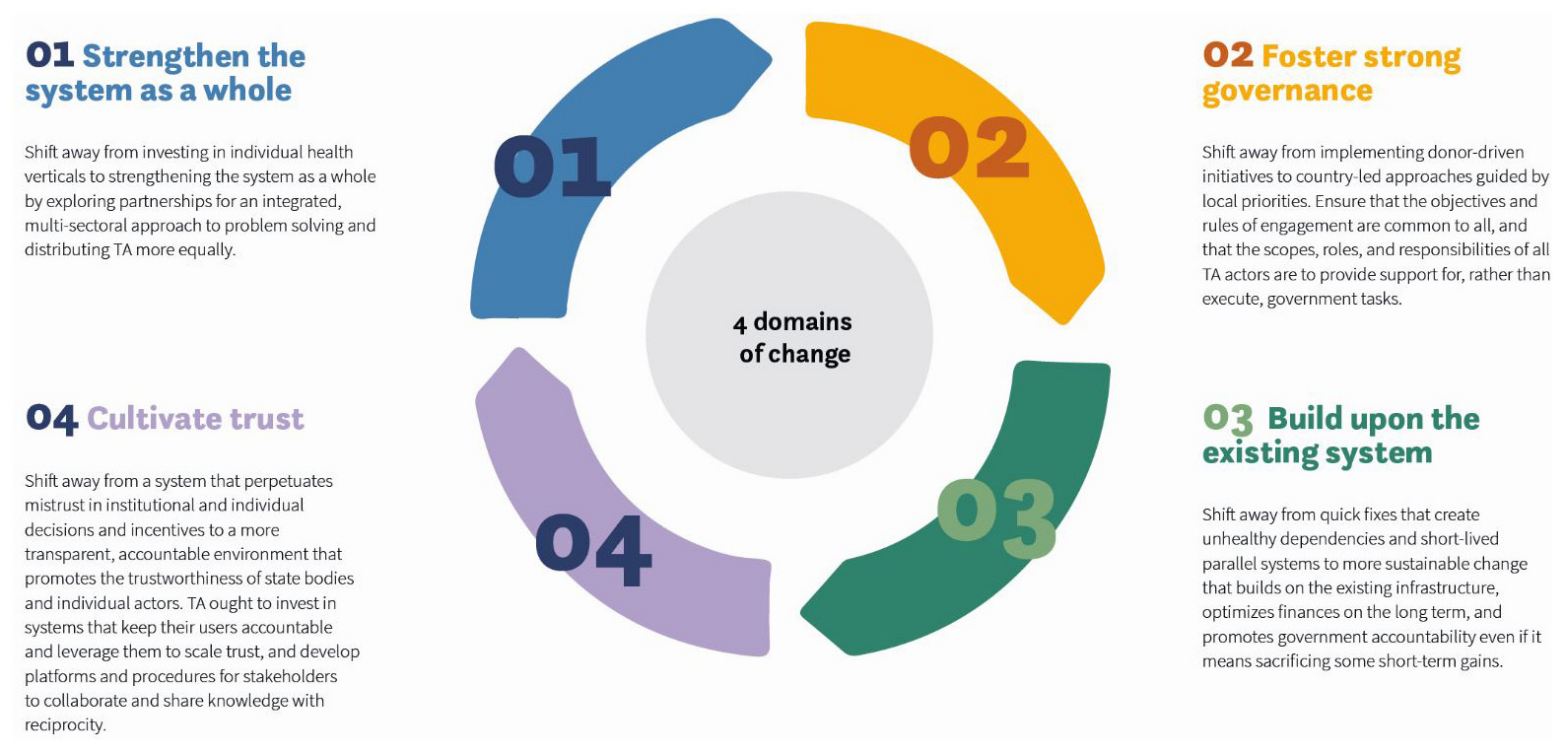
Four domains of change. Note: Has been reproduced with permission from Sonder Collective, 2020 ^22^.

From summaries of the issues that emerged in the interviews, the co-creation teams in the DRC and Nigeria developed design principles of good TA that underpin the necessary shifts and serve as a guide for TA design, engagement, collaboration, and accountability. The nine shifts noted in Table 1 were organized into a framework with four domains of change (i.e., what needs to change). For each domain of change, the co-creation teams identified five design principles. Each principle describes underlying issues and identifies recommended actions to enact change. The 20 principles for good TA are available as extended data to accompany this paper ^25^. The four domains of change are presented in Figure 2. Capacity building is an integral, crosscutting component of all four domains.

In addition to the four domains presented above, the DRC co-creation team emphasized the importance of fostering collaboration and transparency by strengthening governments’ capacities in financial planning, management, and accountability. Specifically, the team recommended the development of platforms and procedures to encourage health system stakeholders to collaborate and share knowledge to build reciprocity in planned and ongoing TA projects, address unmet needs, and leverage opportunities for TA coordination. Furthermore, the DRC co-creation team recommended more robust management of finances, budgets, and incentive schemes to improve the allocation and use of resources. Finally, they emphasized the importance of not lowering governance and accountability standards to continue to advance autonomous health systems management.

## Discussion

The RTA team used a novel approach to exploring issues with TA in the health sector and framing new principles and potential solutions. The project applied HCD techniques, including design research and co-creation of solutions, to uncover insights into the experiences of TA donors, providers, and consumers in Nigeria and the DRC. The team used a country-centric approach, intentionally prioritizing participation by those based in Nigeria and the DRC so that learnings were centered on their experiences and the vision for new approaches to TA were informed by their perspectives.

We drew on an extensive body of interviews and project reports that revealed a detailed description of the limitations of health-sector TA and the factors that contribute to its inefficiencies and ineffectiveness. The project team’s long-term engagement in both countries allowed us to identify emerging themes, confirm or adapt the themes through ongoing reflection with participants, and integrate new questions into subsequent rounds of problem framing and solution generation.

The design research identified several clear shortcomings in how TA is currently planned and implemented and the root causes of the shortcomings. The project team and participants then co-created a set of principles for shifting current global health TA practices toward a more rewarding, effective, and sustainable practice, including related recommended actions and behaviors. This approach to assessing TA differs from other studies of TA effectiveness because it explicitly sought to increase understanding of the TA experience from the perspective of country actors and to generate ideas in collaboration with the actors to reframe TA and devise solutions that address long-standing barriers to its effectiveness.

### Problem framing

The project team identified TA issues similar to those found in other studies of TA, health sector foreign assistance, and health systems strengthening ^26– 31^. Some of the studies are more than 10 years old, which suggests limited effort—or commitment?—among stakeholders to address TA inadequacies and to improve TA processes and effectiveness. Past studies of TA in global health initiatives reveal that despite progress in controlling disease, low- and middle-income country (LMIC) health systems remain fragile because of aid dependency, poor harmonization between government and donor investment priorities, limited improvement in local capacity, and fragmented and parallel data systems for tracking performance in the health sector ^26– 28^. In keeping with TA shortcomings identified in Nigeria and the DRC, MacKellar ^29^, Shiffman ^30^, and Esser and Bench ^31^ document misalignment between country priorities and donor-funded TA projects and inadequate coordination of TA efforts among TA providers and between TA providers and country actors as evidence of the inherent weaknesses of TA as a driver of change.

In contrast to prior studies, RTA’s design research focuses on the perspectives of individuals who provide or receive TA, illustrating through stories and examples the patterns of behavior that characterize TA, the accepted yet faulty mechanisms for implementing TA, the frequent resentment and frustration of all actors with TA processes, and the underlying power differentials that perpetuate a lack of focus on country ownership, sovereignty, and capacity development. TA consumers and providers alike note that in TA relationships it is accepted that donors typically have the power to determine TA agendas, scopes, and the timeframe of interventions and financing cycles, while government actors often feel obligated to accept support as part of the agreement between donor and consumer, even if the support is neither pertinent nor of benefit.

In our research, differential power relationships among TA actors emerged as a strong driver of TA. The institutions and individuals who fund and provide TA influence interactions, priority setting, relationships, and decision making among and within the groups of TA actors. This was echoed by LaFond ^32^, who noted that MOHs are often unable to negotiate the terms of aid and select the programs that are funded due to their reliance on the funding. They prefer to accept the funds and not risk offending the donor and losing the finances. LaFond also explains that donors face a dual dilemma: producing results to present to their funding base (e.g., in the case of government agencies the funding base is tax-paying populations) to ensure continued support for foreign aid, and responding to the priorities of the countries that receive aid. This inherent tension contributes to the misalignment of TA with country priorities.

### Suggested solutions to improve TA

In conceiving solutions to transform TA into a more effective and responsive intervention, country actors in Nigeria and the DRC focused on practical and political steps to address the power imbalance and transfer responsibility for driving health sector improvements to government institutions and actors. They recommend that TA donors, providers, and consumers alike agree to new principles of TA practice that place country needs and institutions at the center of the TA relationship. For example, participants emphasized creating realistic TA timelines and work plans that incorporate sufficient time for those involved in TA to lead, contribute to, coordinate, and implement TA. The concept of inclusive partnerships is also emphasized in the 2008 Accra Agenda, but the fact that it is rarely operationalized remains a significant barrier in today’s TA landscape.

RTA participants emphasized the importance of governments establishing their own agendas and taking the lead in coordinating TA based on country priorities, echoing the recommendations of the Accra Agenda, the Paris Declaration, and the Busan High Level Forum. Similarly, the U.S. Global Health Initiative ^33^ of 2010 identified country ownership as a central strategy for sustainability and accountability. The BMGF- and David and Lucile Packard Foundation-funded 2008 Ministerial Leadership Initiative for Global Health also proposed advancing country ownership through country-led planning and demand-driven TA to ensure that leaders articulate their visions of success and determine country priorities ^34^. RTA participants further reinforced the principles of sustainability, accountability, transparency, and trust among TA donors, providers, and consumers as fundamental to improving TA and stressed that future TA should focus on investing in systems that hold everyone accountable.

Participants noted that governments do not necessarily have the skills or motivation to lead and may not have mechanisms to hold donors and TA providers accountable and to require the alignment of TA to national priorities. Spicer *et al.*’s seven-country study reveals that most countries have several coordination bodies focused on different verticals, topics, and donors—a system that fragments the coordination and monitoring of aid and TA ^35^. A WHO report on improving aid coordination in the health sector ^36^ that compiles lessons from 10 countries, including the DRC, highlights the long-term damage in the DRC from uncoordinated aid and parallel systems for program delivery and data collection that result in overburdened and unmotivated staff.

The challenges of coordinating and managing aid are connected to larger issues of governance not explored in this project that bear surfacing, given their significance. There is extensive literature on the positive association between strong country governance and health outcomes across a range of LMICs ^37– 42^. An analysis by Ruiz-Cantero, Guijarro-Garvi, Bean, Martínez-Riera, and Fernández-Sáez ^43^, examined the association between maternal mortality and country performance based on the World Bank's Worldwide Governance Indicators (i.e., government effectiveness, regulatory quality, rule of law, control of corruption, voice and accountability, and political stability and the absence of violence). The findings suggest that regardless of country wealth, countries that scored higher on the governance indicators had lower levels of maternal mortality. Lin, Chien, Chen, and Chan reported similar findings related to the positive correlation between strong governance and improved child mortality outcomes ^44^.

On the topic of governance, Nigerian RTA participants shared the following example of decentralization, devolution, and state government autonomy to determine their health priorities: in one Nigerian state, donors, TA providers, and governments signed a memorandum of understanding to solidify their partnership and accountability to each other. In doing so, they enhanced mutual trust, committed to reaching alignment on TA goals and priorities, and ensured a seat at the table for the government.

Similar to the critical shifts needed to build health system resiliency, Potter and Brough advocate for a comprehensive approach to capacity building that includes strengthening health systems and infrastructure and skill building to ensure health system sustainability ^16^. They also suggest that TA methods should be regularly evaluated to ensure that they are effective and meet the needs of the populations they are designed to serve. The small number of evaluations of TA quality ^17, 36, 45^ make it difficult to discern what works and what does not, which can lead to the proliferation of ineffective TA practices that hamper progress in system building and improving health outcomes.

### Application of TA principles

The guiding principles defined by RTA participants highlight the need to reframe TA change processes to prioritize a country’s motivation, skills, ownership, and responsibility for improving health systems functions and outcomes. A strong and enduring TA model must include multi-directional leadership, collaboration, and accountability across TA donors, providers, and consumers. The existing global health TA architecture rewards rapid expenditure and disposition of aid resources, often at the expense of longer-term capacity development and shared responsibility for producing lasting change. It is critical to refocus on accountability measures that optimize the use of TA resources and accelerate the impact of external and national investments in health. In addition to new frameworks and metrics, incentives for establishing and maintaining inclusive and respectful TA partnerships are also needed.

Finally, RTA’s approach for understanding participants’ experiences with TA clearly exposes the limitations of TA. Missing from the project’s proposed critical shifts and TA design principles are practical examples of effective TA and positive deviance to guide the development and replication of good TA partnerships and practice. Further scoping of effective TA is needed to validate the proposed principles for transforming TA and offer practical models of desired behaviors and effective mechanisms for long-term capacity building and sustained country ownership.

### Strengths and limitations of this method

Numerous interviews with a diverse group of TA donors, providers, and consumers ensured that a range of perspectives was represented. The authors had access to a vast and comprehensive repository of raw data and reports for analysis. As RTA was implemented solely in the DRC and Nigeria, we caution against generalizing the findings to other settings.

## Conclusions

RTA gathered critical insights from country-based TA actors, who identified significant inadequacies in the way TA is designed and implemented, explicitly calling out the power asymmetry between those who seek funding and TA services and those who provide it. The core finding that emerged is that the global health architecture for TA, external assistance, and partnering require rethinking and reframing. The use of design research and the application of a systems-thinking framework facilitated a deep understanding of the lived experiences of country-based TA actors, who candidly shared their frustrations with TA and visions for how it could be transformed.

RTA frames TA as part of a complex adaptive system, rather than as the mechanistic process it is often characterized as. These findings clearly suggest that TA is not a linear, isolated mechanism that operates in a vacuum, but a complex system engaging a broad network of actors responding and reacting to a wide range of pressures and expectations.

It is critical to increase efforts to assess the effectiveness of TA and identify approaches to guide future TA investments and best practices. While total health spending has increased over the past 20 years, patterns of spending have changed: foreign assistance spending has begun to plateau and spending from country governments and private foundations in LMICs is increasing ^46, 47^. Given these shifting trends, the global health community must learn from past experiences to improve the efficiency and effectiveness of TA in LMICs.

The need for more effective TA planning and delivery is a global challenge, not one that is specific to the DRC and Nigeria. In order for RTA’s findings to be adapted and broadly amplified, actors across the TA ecosystem will need to discuss the implications of the findings, reformulate how TA is designed and implemented, and align as much as possible with the suggestions provided by the DRC and Nigeria participants.

Strengthening TA takes on special urgency when infectious disease outbreaks like Ebola and COVID-19 strain domestic, regional, and global economies and public health systems, and countries have to make difficult decisions about resource allocation and the use of TA services. COVID-19 has highlighted the need for resilient health systems with a capable workforce and sufficient infrastructure for managing routine health service delivery and responding to emergencies. As noted in the United Nations’ Development Cooperation Forum Survey Study 2020, as countries focus on pandemic response and recovery, they will require capacities to marshal, organize, and allocate resources while ensuring the health of their people. It will be essential that capacity-building TA be nimble and responsive to context, and that TA stakeholders invest the time and resources to understand what works and what does not, and evolve accordingly ^48^.

As mentioned in the commentary by Glassman, Chalkidou, and Sullivan, countries cannot all apply the same frameworks for prevention or mitigation due to differences in infrastructure and funding, factors that require strategies customized to local realities ^49^. And, as Cash and Patel stated in a recent piece, “Context is central to any epidemic.” They also advised the global health community to be cognizant of country circumstances before endorsing strategies that have been successful in a select group of countries ^50^. In his article in Global Health NOW, Pai advocates for a reworking of the entire TA architecture beginning with how and where global health professionals are trained, to how TA is funded and who gets invited to the table when funding priorities are set, the required level of expertise of those who provide TA, as well as capacity strengthening approaches to ensure long-term sustainability ^51^.

While there are multiple conceptual frameworks and roadmaps for improving aid effectiveness, there is a dearth of evidence about which combination of TA strategies works in which settings. Future research can test the shifts in TA suggested in this article and identify approaches that are effective in different settings.

## Data availability

### Underlying data

This manuscript drew on text from project reports and interview transcripts. The project reports can be accessed directly from the reference list or in the extended data below. The qualitative transcripts are not openly available for data protection reasons because despite removing identifiable information like names and organizational affiliations, we risk revealing individual identifies through the interview responses. As part of the verbal consent agreement with participants, we assured them of anonymity when presenting synthesized findings. Requests for data must be provided in writing and should include a detailed rationale. All requests must be made by email to Natasha Kanagat at natasha_kanagat@jsi.com. Access may be granted for legitimate research purposes. The John Snow, Inc. IRB will review all requests. 

### Extended data

Zenodo: Country Perspectives on Improving Technical Assistance in the Health Sector. https://doi.org/10.5281/zenodo.5104950 ^25^.

This project contains the following extended data:

- Extended Data - RTA project interview guide.pdf
- Extended Data - RTA project timeline and co-creation phases.pdf
- Extended Data - RTA project twenty principles of good technical assistance.pdf
- Re-Imagining TA for MNCH and Health Systems Strengthening DRC Country Case Study.pdf
- Re-Imagining TA for MNCH and Health Systems Strengthening Nigeria Country Case Study.pdf
- Re-imagining Technical Assistance - Global Design Principles, Nigeria and the Democratic Republic of Congo Case Study.pdf
- Reimagining Technical Assistance - Insights and Opportunities Report.pdf

Data are available under the terms of the Creative Commons Attribution 4.0 International license (CC-BY 4.0).
