# Supplementary material for: Country perspectives on improving technical assistance in the health sector
Source: Gates Open Res. 2021 Aug 24;5:141. [Version 1] doi: 10.12688/gatesopenres.13248.1 (PMC8847213; doi:10.12688/gatesopenres.13248.1)
Supplement: Peer review history

# Peer review: Reviewer response for version 1

**DOI:** 10.21956/gatesopenres.14482.r31237

**Published:** 2022-02-15

**Authors:** Michael Reyes

**Affiliations:** 1 International Training and Education Center for Health, University of California San Francisco, San Francisco, CA, USA; 2 Pacific AIDS Education & Training Center, University of California, San Francisco, CA, USA

An excellent landscape of the quandaries of providing technical assistance - using a two country case study. The authors provide nine critical shifts and four domains of change that have **wide applicability.**

 My big picture observation was that this is an excellent contribution to the global health capacity building literature. The experiences and testimonies detailed **directly** reflect my experience in 20 plus other countries in Africa, Asia and the Caribbean.  I think that the manuscript doesn't really need much improvement and offer my thoughts for consideration only.

 I was interested in more demographic information on interviewees - and found Figure 1 to be vague and not helpful (not unlike a Venn Diagram - unless I viewed it incorrectly). Might you add the number of interviewees in each category?

 This is a complex and nuanced topic, and I thought the authors did an excellent job of categorizing and presenting their background and eventual framework. One thing I was waiting for was the discussion of *power dynamics* that harken back to the home donor institution. It was finally addressed, briefly, in the Discussion Problem Framing: LaFond and the dual dilemma. In my experience this was a primary driver for the TA disconnect. The manuscript might benefit from this discussion coming earlier with a bit more elaboration. My perspective was from PEPFAR and data driven requirements emanating from Washington DC to be implemented at district levels. Disconnects on so many levels requiring, at times, sudden programmatic pivots that made little sense to localities.

 I was also hoping to see more of a discussion of cross government TA engagement that brought in other TA actors such as Ministries of Education (workforce) and Finance (funding for long-term sustainability). I was hoping for a bit more discussion on "embedded TA providers" and the fact that there is often no planning for the end/absorption of the position into government.

 As I read this manuscript, everything ran true, with a nuance or two. The data speaks loudly to these findings. However, in another "vertical" that is PEPFAR, the Country Operation Planning process did try to solve the country level government disconnect with a variety of systems. Though not all implemented, that body of work on community engagement deserves further exploration (along with excellent programmatic technical guidance. Not the scope of this paper). I found the PEPFAR Capacity Building and Strengthening Framework (2014) to be an excellent primer on the types on longstanding outcomes that might be considered to truly build capacity. In my experience, it got very little attention, but I have used that framework for work in the United States.

 Finally, as I read (and reread) this fine manuscript, I pondered its use in Journal Clubs across the world.  What a wonder it would be to have "book club questions" to guide TA actors as they contextualize this in their own countries! (Not the scope of this paper.)

Is the work clearly and accurately presented and does it cite the current literature?

Yes

If applicable, is the statistical analysis and its interpretation appropriate?

Partly

Are all the source data underlying the results available to ensure full reproducibility?

Yes

Is the study design appropriate and is the work technically sound?

Yes

Are the conclusions drawn adequately supported by the results?

Yes

Are sufficient details of methods and analysis provided to allow replication by others?

Partly

Reviewer Expertise:

For 30 years I've worked on HIV multidisciplinary clinical capacity development in the United States. For 17 years I co-directed a health system strengthening project as an implementing partner under PEPFAR in over 25 countries.  I've also worked in the fields of homelessness, preterm birth and transgender health.

I confirm that I have read this submission and believe that I have an appropriate level of expertise to confirm that it is of an acceptable scientific standard.

# Peer review: Reviewer response for version 1

**DOI:** 10.21956/gatesopenres.14482.r31122

**Published:** 2021-10-12

**Authors:** Peter Waiswa

**Affiliations:** 1 Department of Health Policy Planning and Management, Makerere University School of Public Health, Kampala, Uganda; 2 Department of Global Health, Karolinska Institutet, Stockholm, Sweden

This is a very good piece of work. However, the following could be done to improve the work:

1. I saw that the paper is just full of "lamentations". Weren't there any positive results of TA? At least it could be brought in during the discussion e.g. example countries from say Asia where TA is/was positive or evolved to that.
2. I see that almost all problems are put on donors? My thinking (and I am from a developing country), is that national governments are just weak and/or enjoy the chaos - as governments or individuals or the elites in them. To move forward, governments have to lead the change, otherwise we do not expect donors to just do it without government leadership, and pressure.
3. In the table, I suggest a column should be added and in a corresponding text, on who should do what.
4. I would like to see a table summarising the participant characteristics. They only say "donors" and "government workers". As a reader, I would like to know more. Were they technocrats, politicians, etc. What was the mix?

Is the work clearly and accurately presented and does it cite the current literature?

Yes

If applicable, is the statistical analysis and its interpretation appropriate?

Not applicable

Are all the source data underlying the results available to ensure full reproducibility?

Yes

Is the study design appropriate and is the work technically sound?

Yes

Are the conclusions drawn adequately supported by the results?

Yes

Are sufficient details of methods and analysis provided to allow replication by others?

Yes

Reviewer Expertise:

Maternal, newborn, and child health. Also, health systems and policy

I confirm that I have read this submission and believe that I have an appropriate level of expertise to confirm that it is of an acceptable scientific standard.
